# Novel mechanisms of macrolide resistance revealed by *in vitro* selection and genome analysis in *Mycoplasma pneumoniae*

**DOI:** 10.3389/fcimb.2023.1186017

**Published:** 2023-05-22

**Authors:** Na Wang, Xiaogang Xu, Li Xiao, Yang Liu

**Affiliations:** ^1^ Institute of Antibiotics, Huashan Hospital, Fudan University, Shanghai, China; ^2^ Key Laboratory of Clinical Pharmacology of Antibiotics, Ministry of Health, Shanghai, China; ^3^ Department of Medical Oncology, Fudan University Shanghai Cancer Center, Shanghai, China; ^4^ Department of Medicine, University of Alabama at Birmingham, Birmingham, AL, United States

**Keywords:** *Mycoplasma pneumoniae*, macrolides, midecamycin, resistance mechanisms, mutation

## Abstract

*Mycoplasma pneumoniae* is an important pathogen causing upper and lower respiratory tract infections in children and other age groups. Macrolides are the recommended treatments of choice for *M. pneumoniae* infections. However, macrolide resistance in *M. pneumoniae* is increasing worldwide, which complicates the treatment strategies. The mechanisms of macrolide resistance have been extensively studied focusing on the mutations in *23S rRNA* and ribosomal proteins. Since the secondary treatment choice for pediatric patients is very limited, we decided to look for potential new treatment strategies in macrolide drugs and investigate possible new mechanisms of resistance. We performed an *in vitro* selection of mutants resistant to five macrolides (erythromycin, roxithromycin, azithromycin, josamycin, and midecamycin) by inducing the parent *M. pneumoniae* strain M129 with increasing concentrations of the drugs. The evolving cultures in every passage were tested for their antimicrobial susceptibilities to eight drugs and mutations known to be associated with macrolide resistance by PCR and sequencing. The final selected mutants were also analyzed by whole-genome sequencing. Results showed that roxithromycin is the drug that most easily induces resistance (at 0.25 mg/L, with two passages, 23 days), while with midecamycin it is most difficult (at 5.12 mg/L, with seven passages, 87 days). Point mutations C2617A/T, A2063G, or A2064C in domain V of *23S rRNA* were detected in mutants resistant to the 14- and 15-membered macrolides, while A2067G/C was selected for the 16-membered macrolides. Single amino acid changes (G72R, G72V) in ribosomal protein L4 emerged during the induction by midecamycin. Genome sequencing identified sequence variations in *dnaK*, *rpoC*, *glpK*, *MPN449*, and in one of the *hsdS* (*MPN365*) genes in the mutants. Mutants induced by the 14- or 15-membered macrolides were resistant to all macrolides, while those induced by the 16-membered macrolides (midecamycin and josamycin) remained susceptible to the 14- and 15-membered macrolides. In summary, these data demonstrated that midecamycin is less potent in inducing resistance than other macrolides, and the induced resistance is restrained to the 16-membered macrolides, suggesting a potential benefit of using midecamycin as a first treatment choice if the strain is susceptible.

## 1 Introduction

*Mycoplasma pneumoniae* (*M. pneumoniae*) is a small, cell wall-less, and pleomorphic bacterium which belongs to the order Mycoplasmatales, family Mycoplasmataceae, and class Mollicutes (Waites and Talkington, 2004; Waites et al., 2017). It is one of the major mucosal pathogens of the respiratory tract that causes infectious diseases in humans, especially in school-age children and adolescents (Atkinson et al., 2008). Additionally, some researchers have observed that a portion of children typically carry *M. pneumoniae* within the upper respiratory tract asymptomatically (Spuesens et al., 2013; Meyer Sauteur et al., 2016). Under certain conditions, this “atypical” bacterium has also been reported to be responsible for many extrapulmonary manifestations, such as hematologic disorders and central nervous system and dermatological diseases, that can range in severity from mild to life-threatening (Waites et al., 2017).

Because of their lack of a cell wall, *M. pneumoniae* is innately resistant to many classes of antimicrobial agents that act on the cell wall (Waites et al., 2017; Lee et al., 2018). Effective antimicrobials against *M. pneumoniae* include fluoroquinolones (levofloxacin, ciprofloxacin, and moxifloxacin) that inhibit DNA replication and macrolides (erythromycin, azithromycin, and josamycin) and tetracyclines (minocycline and doxycycline) that both inhibit protein synthesis (Waites et al., 2017; Gautier-Bouchardon, 2018). Owing to the potential toxicities of fluoroquinolones and tetracyclines, macrolides are used as the first-line therapy for *M. pneumoniae* infections, particularly in children (Waites et al., 2017). Azithromycin is one of the most commonly used medications throughout the world because of its great tolerance and longer half-life (Waites et al., 2017). However, many studies have reported the increase of macrolide-resistant *M. pneumoniae* (MRMP) strains and related treatment failure worldwide since the early 2000s (Okazaki et al., 2001; Okazaki et al., 2007; Kawai et al., 2012; Kawai et al., 2013; Waites et al., 2017), and over 90% isolates were MRMP in some regions of China and Japan (Liu et al., 2012; Komatsu et al., 2014; Zhou et al., 2015; Wang et al., 2022). Currently, there is no safe and effective secondary treatment option for young children once macrolide resistance develops.

The resistance mechanisms of MRMP have been demonstrated by *in vitro* selection that involves point mutations in the peptidyl transferase loop of *23S rRNA* and point mutations, insertions, or deletions in ribosomal proteins L4 and L22 (Lucier et al., 1995; Pereyre et al., 2004; Ou et al., 2015; Waites et al., 2017). Some of these changes were observed in clinical MRMP isolates (Morozumi et al., 2005; Xin et al., 2009; Wang et al., 2019), while some were not (Pereyre et al., 2004; Ou et al., 2015), probably because these MRMP strains were induced by subminimum inhibitory concentrations of macrolide antibiotics. The investigation of macrolide resistance mechanisms in *M. pneumoniae* has mainly been focused on the single *23S RNA* gene and ribosomal protein genes, and so further analysis on other potential resistance mechanisms is required. To better understand macrolide resistance in *M. pneumoniae* and make better usage of currently available macrolide drugs, we performed an *in vitro* study to select macrolide-resistant mutants in the parent strain by using increasing concentrations of five macrolides and characterized the final mutants by whole-genome sequencing.

## 2 Materials and methods

### 2.1 Bacterial strains, growth conditions, and antibiotics

The macrolide-susceptible reference strain *M. pneumoniae* M129 (ATCC 29342) was used as the parent strain to select macrolide-resistant mutants. Before induction, M129 colonies were clone-purified and inoculated at 37°C in mycoplasma broth for 5–7 days until a color change (pink to orange-yellow) occurred. The cultures were then aliquoted and stored at −70°C. As previously reported by Sun et al., mycoplasma broth was prepared using mycoplasma broth base CM403 (Oxoid, Hampshire, United Kingdom), mycoplasma selective supplement G SR59 (Oxoid), 0.002% phenol red, and 0.5% glucose. Mycoplasma agar plates, containing mycoplasma agar base CM401 (Oxoid) and mycoplasma selective supplement G SR59, were also prepared (Sun et al., 2008). Five macrolides and three other drugs were included in the study. Erythromycin and roxithromycin as 14-membered ring macrolides were purchased from the National Institutes for Food and Drug Control (Shanghai, China). Azithromycin (15-membered ring macrolide) and josamycin and midecamycin (16-membered ring macrolides) were purchased from Dalian Meilun Biotechnology Co., Ltd. (Dalian, China). Tetracycline and moxifloxacin were also from the National Institutes for Food and Drug Control (Shanghai, China). Nemonoxacin was provided by Zhejiang Medicine Co., Ltd. (Xinchang, China).

### 2.2 Selection of macrolide-resistant *Mycoplasma pneumoniae* mutants and mutation detection

The *in vitro* selection of macrolide-resistant mutants was conducted according to the previously described methods with minor modifications (Reinhardt et al., 2002; Tatay-Dualde et al., 2017). Briefly, the minimum inhibitory concentrations (MICs) of the parent strain to the five macrolides were determined (Waites et al., 2011). The induction inocula contained 1 ml of organisms (approximately 5 × 10^4^ CFU/ml) and each macrolide drug at four concentrations in a two-fold increasing step (0.00064, 0.00128, 0.00256, and 0.00512mg/L) initially (first step). When the medium color changed from red to yellow, approximately 0.1 ml of the culture from the tubes with the most concentrated drugs was transferred into a new culture tube containing the next serial concentrations of the drugs (starting from the highest concentration showing color change from the first step). The remaining cultures were aliquoted and stored at −80°C for further analysis. One aliquot was processed for antimicrobial susceptibility testing and one was used for PCR assays. The selection step was repeated as described above until the inducted strain was confirmed resistant to the macrolide of induction by antimicrobial sensitivity testing.

The point mutations in the *23S rRNA* gene were detected by nested PCR using a previous method (Sun et al., 2013). The ribosomal protein genes *rplD* (L4) and *rplV* (L22) were amplified using primer pairs described previously (Pereyre et al., 2004). Sanger sequencing was performed on the amplicons, and sequences were compared with those of the reference strain.

### 2.3 Antimicrobial susceptibility test

The MICs of eight drugs consisting of five macrolides, two quinolones, and tetracycline were determined using the broth microdilution method, according to the recommendation of the CLSI (Waites et al., 2011).

### 2.4 Whole-genome sequencing and sequence variation detection

Genomic DNA from 50 ml of the end selected culture and the parent strain was extracted using the TIANamp Bacteria DNA Kit (DP302) from Tiangen Biochemical Technology (Beijing) Co., Ltd., China, according to the manufacturer’s instructions. Genome sequencing was performed by Shanghai Yuanxu Biotechnology Co., Ltd., China. Briefly, genomic DNA was sheared using KAPA Frag Kit for Enzymatic fragmentation (KK8600, Kapa Biosystems, Wilmington, MA, USA), and sequencing libraries were constructed *via* VAHTS Universal DNA Library Prep Kit for Illumina V4 (ND610-02, Vazyme, Nanjing, China). Pair-end libraries were sequenced on HiSeq X Ten platforms with a read length of 150 bp. Genome reads were deposited to the Sequence Read Archive (SRA, BioProject ID PRJNA954979).The average number of reads per sample was 7,178,870 with more than 200× coverage on average. Sequences were analyzed using CLC Genomics Workbench 23 (Qiagen, Valencia, CA, USA). *Mycoplasma pneumoniae* M129-B7 reference chromosome (GenBank accession number NC_020076) was used to map the reads, and sequence variations, including single nucleotide variants (SNVs), insertions, deletions, and replacements, were detected using the Basic Variant Detection tool. The resulting sequence variations in the mutants were then compared with that of the parent strain and differences were recorded.

## 3 Results

### 3.1 Selection of macrolide-resistant mutants

The parent reference strain M129 was susceptible to all drugs tested, with MICs equal to or lower than 0.015 mg/L for the five macrolides and 0.125 mg/L for quinolones and tetracycline (**Table 1**). Mutants resistant to macrolides were successfully selected by serial passages of the parent strain in the increasing concentrations of erythromycin, roxithromycin, azithromycin, josamycin, and midecamycin. The induction took two to seven passages or 23–87 days for different macrolides. Changes of the MICs and the emergence of mutations in the *23S rRNA* gene and *rplD* were observed during the passages (**Table 1**). The MICs of the final selected mutants were increased 1,067- to 160,000-fold compared with the parent strain. The selected mutants displayed different MIC characters for the five macrolides, while no changes for quinolones and tetracycline were observed.

**Table 1:** Characteristics of macrolide-resistant *Mycoplasma pneumoniae* mutants collected at each selection step.

Macrolides (total induction days)	Passages	Incubation days	Concentration (mg/L)	Mutations			MICs (mg/L)							
				*23S rRNA*	*rplD*	*rplV*	ERY	ROX	AZI	JOS	MID	NEM	MOX	TET
–	Parent strain	–	–	–	–	–	0.0025	≤0.00375	0.0002	0.015	0.015	0.125	0.125	0.125
ERY (52 days)	E1	5	0.00064	–	–	–	≤0.06	≤0.06	≤0.06	≤0.06	≤0.06	0.125	0.125	0.125
	E2	4	0.00512	–	–	–	≤0.06	≤0.06	≤0.06	≤0.06	≤0.06	0.125	0.125	0.125
	E3	9	0.0256	–	–	–	≤0.06	≤0.06	≤0.06	≤0.06	≤0.06	0.125	0.125	0.125
	E4	21	0.1024	C2617A	–	–	0.125	0.5	≤0.06	≤0.06	≤0.06	0.125	0.06	0.125
	E5	13	0.64	C2617A, A2063G	–	–	>128	>128	128	64	32	0.125	0.125	0.125
ROX (23 days)	R1	5	0.00512	–	–	–	≤0.06	≤0.06	≤0.06	≤0.06	≤0.06	0.125	0.125	0.125
	R2	18	0.25	A2064C	–	–	>128	>128	4	64	128	0.25	0.25	0.125
AZI (58 days)	A1	11	0.00128	–	–	–	≤0.06	≤0.06	≤0.06	≤0.06	≤0.06	0.125	0.125	0.125
	A2^a^	26	0.00512	C2617T	–	–	–	–	–	–	–	–	–	–
	A3^a^	15	0.0512	C2617A	–	–	–	–	–	–	–	–	–	–
	A4	6	0.512	A2063G	–	–	>128	>128	32	4	4	0.125	0.125	0.25
JOS (28 days)	J1	5	0.005	–	–	–	≤0.06	≤0.06	≤0.06	≤0.06	≤0.06	0.125	0.125	0.125
	J2	23	1.024	A2067G	–	–	≤0.06	0.125	≤0.06	16	64	0.125	0.125	0.125
MDI (87 days)	M1	5	0.00512	–	–	–	≤0.06	≤0.06	≤0.06	≤0.06	≤0.06	0.125	0.125	0.125
	M2	7	0.032	–	–	–	≤0.06	≤0.06	≤0.06	0.125	≤0.06	0.125	0.125	0.125
	M3	17	0.128	–	G214A (G72R)	–	≤0.06	0.125	≤0.06	0.5	0.5	0.125	0.125	0.125
	M4	9	0.128	–	G214A (G72R)	–	≤0.06	0.125	≤0.06	0.5	0.5	0.125	0.125	0.125
	M5	18	0.256	–	G214A (G72R)	–	0.125	0.25	≤0.06	0.5	0.5	0.125	0.125	0.125
	M6	12	0.512	–	G215T (G72V)	–	0.125	0.25	≤0.06	1	0.5	0.125	0.125	0.125
	M7	19	5.12	A2067C	G214A (G72R)	–	0.125	0.125	≤0.06	32	64	0.125	0.125	0.125

ERY, erythromycin; ROX, roxithromycin; AZI, azithromycin; JOS, josamycin; MID, midecamycin; TET, tetracycline; MOX, moxifloxacin; NEM, nemonoxacin.

a Failure to resuscitate the strains in the second and third passages of induced resistance by azithromycin. "-" in column "Mutations" means "No mutation detected"; in column (MICs) means "Not available".

#### 3.1.1 Stepwise evolution toward 14-membered ring macrolide resistance

The selection of erythromycin-resistant mutants took 52 days with five passages (**Table 1**). Resistance and mutations were not detected in the first three passages under the influence of erythromycin concentrations equal to or lower than 0.0256 mg/L (E1–3). At the fourth passage (E4), mutation C2617A in the *23S rRNA* gene emerged, accompanied by slightly increased MICs for erythromycin and roxithromycin (0.125 and 0.5 mg/L). At the fifth passage (E5), A2063G in *23S rRNA* emerged in addition to C2617A, and MICs were increased for all macrolides (>128 mg/L for erythromycin and roxithromycin, 128 mg/L for azithromycin, 64 mg/L for josamycin, and 32 mg/L for midecamycin). The selection of roxithromycin-resistant mutants was the quickest, only taking 23 days and two passages (**Table 1**). At the second passage (R2), mutation A2064C in the *23S rRNA* gene appeared, and MICs were also increased for all macrolides (>128 mg/L for erythromycin and roxithromycin, 4 mg/L for azithromycin, 64 mg/L for josamycin, and 128 mg/L for midecamycin).

#### 3.1.2 Stepwise evolution toward 15-membered ring macrolide resistance

The selection of azithromycin-resistant mutants took 58 days and four passages (**Table 1**). Unfortunately, frozen culture aliquots from the second and third passages (A2 and A3) failed to be recovered, and their MICs were not available for them. However, the *23S rRNA* mutations C2617T were detected in passage 2 and C2617A in passage 3. In the fourth passage (A4), A2063G substitution was detected, and increased MICs to all macrolides were observed (>128 mg/L for erythromycin and roxithromycin, 32 mg/L for azithromycin, and 4 mg/L for josamycin and midecamycin).

#### 3.1.3 Stepwise evolution toward 16-membered ring macrolide resistance

The selection of josamycin-resistant mutants was quick, taking 28 days and two passages (**Table 1**). At the second passage (J2), mutation A2067G in the *23S rRNA* gene emerged, and MICs were increased for the 16-membered macrolides josamycin (16 mg/L) and midecamycin (64mg/L). Interestingly, MICs for the 14- and 15-membered macrolides were still low (≤0.125 mg/L).

The selection of midecamycin-resistant mutants took the longest induction time (87 days), the greatest number of passages (seven), and the highest induction drug concentration (5.12 mg/L). Resistance and mutations were not detected in the first two passages (M1 and M2). From the third to the seventh passages (M3–M7), different single point mutations in ribosomal protein L4 gene *rplD* were detected sequentially (nucleotide changes: G214A in M3–M5 and M7, G215T in M6). Slightly increased MICs for midecamycin (0.5 mg/L) and josamycin (0.5 or 1.0 mg/L) were observed in passages M3 to M6. In the last passage (M7), mutation A2067C in the *23S rRNA* gene emerged in addition to the L4 mutation, and the corresponding MICs for josamycin and midecamycin were increased to 32 and 64 mg/L, respectively. Similar to josamycin, the MIC values for the 14- and 15-membered macrolides also remained low (≤0.125 mg/L).

### 3.2 Genome-wide analysis of selected mutants

The genomes of the parent strain M129 and the five selected mutants were partially assembled, and each genome contained 5–13 contigs. The genome size of the parent strain M129 was 816,516 bp, and the range of the five selected mutants was 813,065 to 815,897 bp, with a GC content of approximately 40% for all genomes (**Supplementary Table 1**). Whole-genome alignment analysis revealed that they were highly similar with the average nucleotide identity (ANI) ≥99.99% and alignment percentage (AP) ≥97.15%.

There were 7–11 sequence variations per genome in the final selected mutants (**Table 2**). The variations were distributed in 15 genes and four intergenic regions. Mutations previously detected by PCR in the *23S rRNA* gene and *rplD* were also identified by the genome-wide variant detection. Non-synonymous sequence variations were found in 12 protein-coding genes, namely, *dnaK* (encodes a chaperone protein Hsp70, also named *MPN434* in genome NC_000912), *rpoC* (encodes DNA-directed RNA polymerase beta, *MPN515*), *glpK* (encodes glycerol kinase, *MPN050*), *C985_RS03055* (encodes L-ribulose-5-phosphate 4-epimerase, *MPN498*), *C985_RS02085* (encodes type I restriction-modification system, specificity subunit S, *MPN365*), *C985_RS03730* (encodes YitT family protein, *MPN657*), *C985_RS01220* (encodes DUF5426 family protein, *MPN212*), and four hypothetical protein genes (**Table 2**). There was a G997A SNV identified in the *dnaK* gene that resulted in amino acid change V333M in the mutant induced by azithromycin (A4). BLAST results showed that V333 is conserved in DnaK protein in mycoplasmas and other bacteria (including *Escherichia coli*) and is located in a helix according to the predicted secondary structure. In the mutant induced by erythromycin (E5), SNV G2761A in the *rpoC* gene which caused amino acid change V921M was also identified. The amino acid residue at this position is not conserved among the *Mollicutes* and other bacteria, and V921 is only present in *M. pneumoniae* and *M. genitalium*. It is interesting to observe that in the parent strain, *glpK* (*MPN050*) and *C985_RS03055* (*MPN498*) existed as a mixture carrying both SNVs [C710A (T237N) in *glpK* and C352T (L118F) in *C985_RS03055*] and wild-type sequences (SNV frequency were 36.74% and 50.51%, respectively). After drug induction, the SNVs were selected to be almost pure in all mutants, except for R2 which was totally selected back to the wild-type sequence. There was a 12-bp tandem repeat sequence insertion in one of the *hsdS* genes *C985_RS02085* (*MPN365*) in the mutant induced by roxithromycin R2. Notably, this insertion was detected in 42.19% of the reads, indicating that a dynamic evolution is still ongoing. It is also interesting to find that C985_RS02560 (*MPN449*), which encodes a hypothetical membrane protein, developed four different SNVs in the five mutants (**Table 2**).

**Table 2:** Genetic alterations of genes in mutants.

Mutant	Region	Type	Reference	Allele	Gene name/Locus Tag	Gene alteration	Amino acid alteration	Function
Parent	62792	SNV	G	T	*glpK*	C710A	T237N	Glycerol kinase (EC 2.7.1.30)
Parent	605306	SNV	C	T	C985_RS03055	C352T	L118F	L-ribulose-5-phosphate 4-epimerase
R2	122095	SNV	A	C	C985_RS00540	A2064G		23S rRNA
R2	167971	SNV	C	T	C985_RS00770	C374T	S125L	Hypothetical protein
R2	436118^436119	Insertion	–	CCGAGCTAAGCG	C985_RS02085	Insertion 454GAGCTAAGCGCC	152ELSA	Type I restriction-modification system, specificity subunit S
R2	497605	SNV	C	A				Intergenic region (C985_RS02375 and C985_RS04390)
R2	547563	SNV	A	G	C985_RS02560	T1172C	I391T	Hypothetical protein
R2	605306	SNV	C	T	C985_RS03055	C352T	L118F	L-ribulose-5-phosphate 4-epimerase
R2	622660.622661	Deletion	TT	–				Intergenic region (between C985_RS02925 and C985_RS02930)
E5	62792	SNV	G	T	*glpK*	C710A	T237N	Glycerol kinase (EC 2.7.1.30)
E5	122094	SNV	A	G	C985_RS00540	A2063G		23S rRNA
E5	122648	SNV	C	A	C985_RS00540	C2617A		23S rRNA
E5	195426	Deletion	A	–				Intergenic region (between C985_RS04175 and C985_RS00880)
E5	528862^528863	Insertion	–	T				Intergenic region (between C985_RS02495 and C985_RS02500)
E5	548224	SNV	G	T	C985_RS02560	G511A	R171S	Hypothetical protein
E5	605306	SNV	C	T	C985_RS03055	C352T	L118F	L-ribulose-5-phosphate 4-epimerase
E5	629581	SNV	C	T	*rpoC*	G2761A	V921M	DNA-directed RNA polymerase beta’ subunit (EC 2.7.7.6)
E5	782762	SNV	C	G	C985_RS03730	G380C	R127P	YitT family protein
A4	62792	SNV	G	T	*glpK*	C710A	T237N	Glycerol kinase (EC 2.7.1.30)
A4	122094	SNV	A	G	C985_RS00540	A2063G		23S rRNA
A4	522684	SNV	C	T	*dnaK*	G997A	V333M	Chaperone DnaK
A4	538445	SNV	C	T	C985_RS02535	G3351A	Synonymous	DUF3713 domain-containing protein
A4	547899	SNV	C	A	C985_RS02560	G836T	S279I	Hypothetical protein
A4	605306	SNV	C	T	C985_RS03055	C352T	L118F	L-ribulose-5-phosphate 4-epimerase
A4	782783	SNV	G	A	C985_RS03730	C359T	S120F	YitT family protein
J2	62792	SNV	G	T	*glpK*	C710A	T237N	Glycerol kinase (EC 2.7.1.30)
J2	122098	SNV	A	G	C985_RS00540	A2067G		23S rRNA
J2	497605	SNV	C	A				Intergenic region (C985_RS02375 and C985_RS04390)
J2	497625	SNV	A	C	C985_RS04390	A17C	Stop to S6	Hypothetical protein
J2	497629.497630	MNV	AA	CG	C985_RS04390	A21C, A22G	W7C, R8G	Hypothetical protein
J2	497634	SNV	G	C	C985_RS04390	G26C	R9P	Hypothetical protein
J2	497638	SNV	A	C	C985_RS04390	A30C	Synonymous	Hypothetical protein
J2	527357	SNV	C	G	C985_RS02495	G1311C	Synonymous	DUF3713 domain-containing protein
J2	528862^528863	Insertion	–	T				Intergenic region (between C985_RS02495 and C985_RS02500)
J2	548224	SNV	G	T	C985_RS02560	C511A	R171S	Hypothetical protein
J2	605306	SNV	C	T	C985_RS03055	C352T	L118F	L-ribulose-5-phosphate 4-epimerase
M7	62792	SNV	G	T	*glpK*	C710A	T237N	Glycerol kinase (EC 2.7.1.30)
M7	122098	SNV	A	C	C985_RS00540	A2067C		23S rRNA
M7	219011	SNV	G	A	*rplD*	G214A	G72R	LSU ribosomal protein L4p (L1e)
M7	261605	SNV	G	A	C985_RS01220	G25A	V9I	DUF5426 family protein
M7	497605	SNV	C	A				Intergenic region (C985_RS02375 and C985_RS04390)
M7	497625	SNV	A	C	C985_RS04390	A17C	Stop to S6	Hypothetical protein
M7	528862^528863	Insertion	–	T				Intergenic region (between C985_RS02495 and C985_RS02500)
M7	547423	SNV	C	G	C985_RS02560	G1312C	D438H	Hypothetical protein
M7	605306	SNV	C	T	C985_RS03055	C352T	L118F	L-ribulose-5-phosphate 4-epimerase
M7	781991	SNV	G	A	C985_RS03730	C1151T	S384L	YitT family protein

SNV, single nucleotide variation; MNV, multiple nucleotide variation. "-" means "Not available".

## 4 Discussion

We have successfully selected resistant mutants of *M. pneumoniae* to five different macrolides by *in vitro* induction with increasing concentrations of the drugs. Mutations in *23S rRNA* and ribosomal protein L4 emerged during the passages with corresponding changes in MICs. Midecamycin was the least potent drug to induce resistance. The 14- and 15-membered macrolides induced mutations at position 2063 or 2064 in *23S rRNA*, while the 16-membered macrolides selected mutations at position 2067. Genome sequencing revealed that additional SNVs were developed in other protein-coding genes and intergenic regions in the final selected mutants. Induced mutants resistant to 14- and 15-membered macrolides were cross-resistant to 16-membered macrolides, while those resistant to 16-membered macrolides were still susceptible to 14- and 15-membered ones.

This study showed that the potential of the five macrolides to induce resistance in *M. pneumoniae* was different. Roxithromycin, azithromycin, and josamycin selected only one final mutation in *23S rRNA*, while erythromycin and midecamycin finally selected two mutations in *23S rRNA* or the L4 protein. Roxithromycin, josamycin, and azithromycin induced an initial *23S rRNA* substitution quickly at the second passage. In contrast, erythromycin induced the first mutation in the fourth passage. This result is different from the previous selection study where erythromycin selected mutations earlier and josamycin later (Pereyre et al., 2004), probably due to the different induction strategies used in this study. Midecamycin induced an initial mutation in the L4 protein in passage 3. However, the complete induction of resistance took the most passages and induction days (seven passages, 87 days) and the highest final induction concentration (5.12 mg/L), making it the macrolide most difficult to induce resistance. To our knowledge, this is the first *in vitro* selection of midecamycin resistance in mycoplasmas.

Midecamycin is a naturally occurring 16-membered macrolide synthesized by *Streptomyces mycarofaciens* (Cong and Piepersberg, 2007; Arsic et al., 2018). It is active against the strains susceptible or resistant to erythromycin (Schlegel et al., 2001). This antimicrobial and its derivatives are also active against other bacteria and mycoplasma species and have been widely used in the clinical treatment of respiratory tract infections (Ishida et al., 1994; Wang et al., 2020; Lin et al., 2021). The dosage and safety of midecamycin have already been determined in pediatric patients in different countries. Since azithromycin has been the empirical treatment option for *M. pneumoniae* infections in children, increasing resistance has appeared worldwide in the last 20 years (Waites et al., 2017). Finding an alternative treatment for infections caused by *M. pneumoniae* is urgently needed. The results of this study indicated that it is more difficult to select mutants by midecamycin than by other macrolides. Furthermore, even in the case that the strain developed resistance to midecamycin, it is still susceptible to the 14- and 15-membered macrolides. Taken together, these features of midecamycin suggest that it is a promising alternative first choice of antimicrobial agent for the treatment of mycoplasma infections, especially when susceptible strains are present. This treatment strategy may help reduce the occurrence of macrolide resistance and reserve the 14- and 15-membered macrolides as secondary treatment options. Further clinical trials are needed to clarify their efficacies for this purpose.

Mutations A2063G and A2064C in *23S rRNA* in the mutants induced by the 14- and 15-membered macrolides were the same as the naturally occurring mutations in clinical MRMP strains (Lucier et al., 1995; Bébéar et al., 2011; Pereyre et al., 2016). Mutation C2617A in *23S rRNA* was also described in the previous *in vitro* induction study with a subinhibitory concentration of azithromycin (Pereyre et al., 2004; Bebear and Pereyre, 2005). Josamycin induced the mutation A2067G in *23S rRNA*, which is the same as the other *in vitro* studies in *M. pneumoniae* and *M. hominis* (Furneri et al., 2001; Pereyre et al., 2004) and in an *M. genitalium* clinical strain from failed josamycin treatment (Guschin et al., 2015). This study also reported the first finding of the mutations associated with midecamycin resistance in mycoplasmas. Midecamycin induced mutation A2067C in *23S rRNA* and different mutations in L4 protein [G214A (G72R) and G215T (G72V)]. Interestingly, mutants with A2067 alterations in *23S rRNA* (J2 and M7) were resistant to the 16-membered macrolides, while the remaining were susceptible to the 14- and 15-membered macrolides. This observation is similar to the previous reports on mycoplasmas (Furneri et al., 2001; Pereyre et al., 2004) and in *Streptococcus pneumoniae* (Depardieu and Courvalin, 2001). The different MIC profile probably reflects the difference in binding site, drug orientation, and binding kinetics between the 16-membered macrolides and the 14- and 15-membered macrolides (Starosta et al., 2010). Midecamycin is the only macrolide that selected mutations both in *23S rRNA* and in ribosomal protein L4. The L4 mutations were located close to a previously reported mutation A209T (H70R) that occurred in *in vitro* selected isolates as well as in clinical isolates (Pereyre et al., 2004; Cao et al., 2010; Ou et al., 2015). Mutants harboring only L4 mutations showed slightly decreased susceptibility to the 16-membered macrolides with MICs ≤1 mg/L and did not cause resistance to the 14- and 15-membered macrolides.

This study used whole-genome sequencing to investigate the potential novel mechanisms of macrolide resistance on the mutants selected *in vitro* and identified sequence variations in 15 genes and four intergenic regions. DnaK is a molecular chaperone with multiple functions, including stress response, protein folding, and interacting with the host extracellular matrix in *M. pneumoniae* (Hagemann et al., 2017). It is reasonable to speculate that DnaK is involved in the drug-induced stress response. However, the biological function of the V333M mutation in DnaK could not be ruled out in this study because of the co-occurrence of other mutations related to macrolide resistance. The V921M alteration in the RpoC may not be essential since the V921 residue is not conserved. Another interesting finding is the insertion of a 12-bp tandem repeat unit in one of the *hsdS* genes *MPN365* in mutant R2. Although tandem repeat variation in the *hsdS* genes was predicted to be related to the epigenetic regulation of gene function/expression (Xiao et al., 2015), this is the first observation of *hsdS* variation with the corresponding phenotypic change. The exact role of *hsdS* genes in macrolide resistance development warrants further investigation. The increased percentages of the population harboring SNVs in *glpK* (*MPN050*) and *C985_RS03055* (*MPN498*) with the drug selection indicates that these SNVs favor the adaptation of *M. pneumoniae* to the drug pressures. *glpK* is an essential gene for *M. pneumoniae* with a known function in glycerol metabolism (Hames et al., 2009). This study reveals its possible secondary function in drug resistance. It has been reported that mutations in the *glpK* gene contributed to drug tolerance in *Mycobacterium tuberculosis* and *Mycobacterium bovis* (Safi et al., 2019; Dong et al., 2022). MPN449 has predicted transmembrane structures according to CLC Genomics and the computed structure model from AlphaFold DB (AF_Q50363F1) and I-TASSER (Zheng et al., 2021). Its *M. genitalium* homolog MG_314 was annotated as putrescine transporter PotE (Yang et al., 2020). Two of the amino acid changes (S279I and I391T) in the mutants induced by azithromycin (A4) and roxithromycin (R2) were in the predicted membrane coil and strand, and the other two (R171S and D438H) were in the predicted helix and coil in the cytoplasm. It would be interesting to further investigate the biological function of this protein and its role in macrolide resistance. Although the mechanisms of resistance are unknown for the mutations identified in this study, we expect that they would impose potential fitness costs to *M. pneumoniae*, affecting the growth rate of the mutants in conditions without drugs. However, this study was not set up for this measurement to answer this question.

This study has some limitations. First, although resistance is not expected to occur in the parent strain, a passage control was not included in each selection step, and thus, second, background sequence variation could not be ruled out without the passage controls of the final selected mutants when conducting the variant detection. Third, the drug selection was only performed in a single set without biological replicates, which limits the confidence in determining the induction potential of each drug and the chances of catching more mutations. However, these limitations do not affect the conclusions of this study.

In summary, this study presented the first *in vitro* data of induced midecamycin resistance in *M. pneumoniae* and the potential advantage of using midecamycin as an alternative first treatment choice for *M. pneumoniae* infections in patients.

## Data availability statement

The datasets presented in this study can be found in online repositories. The names of the repository/repositories and accession number(s) can be found below: NCBI, SRA, PRJNA954979.

## Author contributions

YL and LX conceived and designed the experiments. NW performed the experiments. NW, XX, and LX analyzed the data. NW wrote the draft of the manuscript. YL and LX reviewed and edited the manuscript. All authors contributed to the article and approved the submitted version.
