# Supplementary material for: Novel mechanisms of macrolide resistance revealed by in vitro selection and genome analysis in Mycoplasma pneumoniae
Source: Front Cell Infect Microbiol. 2023 May 22;13:1186017. doi: 10.3389/fcimb.2023.1186017 (PMC10240068; doi:10.3389/fcimb.2023.1186017)
Supplement: Supplementary file 1 [file Table_1.docx]

Supplementary Table 1. General genome features of the mutants and the parent strain M129

|  | M129 | E5 | R2 | A4 | J2 | M7 |
| --- | --- | --- | --- | --- | --- | --- |
| Genome size (bp) | 816,516 | 814,834 | 813,966 | 813,065 | 815,897 | 815,428 |
| genes | 1,425 | 1,456 | 1,476 | 1,450 | 1,435 | 1,472 |
| GC (%) | 40.87 | 40.63 | 40.56 | 40.68 | 40.84 | 40.66 |
